# The effect of resolvin D1 on bone regeneration in a rat calvarial defect model

**DOI:** 10.1002/term.3345

**Published:** 2022-08-18

**Authors:** Xiaofeng Jiang, Jing Liu, Si Li, Yingfei Qiu, Xiaoli Wang, Xiaoli He, Torbjørn Ø. Pedersen, Kamal Mustafa, Ying Xue, Manal Mustafa, Alpdogan Kantarci, Zhe Xing

**Affiliations:** ^1^ School/Hospital of Stomatology Lanzhou University Lanzhou China; ^2^ Department of Clinical Dentistry Faculty of Medicine University of Bergen Bergen Norway; ^3^ Department of Maxillofacial Surgery Haukeland University Hospital Bergen Norway; ^4^ Oral Health Centre of Expertise in Western Norway Bergen Norway; ^5^ The Forsyth Institute Cambridge Massachusetts USA; ^6^ Harvard University School of Dental Medicine Boston Massachusetts USA; ^7^ Key Laboratory of Dental Maxillofacial Reconstruction and Biological Intelligence Manufacturing Lanzhou University Lanzhou China; ^8^ Institute of Medical Biology Faculty of Health Sciences UiT The Arctic University of Norway Tromsø Norway

**Keywords:** osteogenesis, resolvin D1, vascularization

## Abstract

Resolvin D1 (RvD1) is a pro‐resolving lipid mediator of inflammation, endogenously synthesized from omega‐3 docosahexaenoic acid. The purpose of this study was to investigate the effect of RvD1 on bone regeneration using a rat calvarial defect model. Collagen 3D nanopore scaffold (COL) and Pluronic F127 hydrogel (F127) incorporated with RvD1 (RvD1‐COL‐F127 group) or COL and F127 (COL‐F127 group) were implanted in symmetrical calvarial defects. After implantation, RvD1 was administrated subcutaneously every 7 days for 4 weeks. The rats were sacrificed at weeks 1 and 8 post‐implantation. Tissue samples were analyzed by real‐time reverse transcriptase‐polymerase chain reaction and histology at week 1. Radiographical and histological analyses were done at week 8. At week 1, calvarial defects treated with RvD1 exhibited decreased numbers of inflammatory cells and tartrate‐resistant acid phosphatase (TRAP) positive cells, greater numbers of newly formed blood vessels, upregulated gene expression of vascular endothelial growth factor and alkaline phosphatase, and downregulated gene expression of receptor activator of nuclear factor‐κB ligand, interleukin‐1β and tumor necrosis factor‐α. At week 8, the radiographical results showed that osteoid area fraction of the RvD1‐COL‐F127 group was higher than that of the COL‐F127 group, and histological examination exhibited enhanced osteoid formation and newly formed blood vessels in the RvD1‐COL‐F127 group. In conclusion, this study showed that RvD1 enhanced bone formation and vascularization in rat calvarial defects.

## 1 INTRODUCTION

Tumors, trauma, and congenital disorders can cause substantial facial bone defects where functional and esthetic reconstruction can be challenging (Li & Li, 2005). Autologous bone grafting is the gold standard in regeneration; however, this approach has disadvantages, including additional operative time for graft harvesting, limitations in bone volume and donor site morbidity (Rogers & Greene, 2012). Alternative bone substitutes such as allografts and xenografts require the removal of organic components due to the possibility of introducing immune rejection and disease transmission. In addition, these treatments result in limited osteoinductive properties, making allo‐ and xenografts less viable options for reconstructing larger defects (Fernandez de Grado et al., 2018). Therefore, bone tissue engineering (BTE) has emerged as an alternative approach to autologous bone grafting.

Resolvins are a class of anti‐inflammatory bioactive lipid mediators generated from omega‐3 eicosapentaenoic acid and docosahexaenoic acid (DHA), denoted E series (RvE) and D series (RvD) resolvins, respectively (Norling & Serhan, 2010). Resolvins promote the resolution of inflammatory responses by controlling neutrophil recruitment and modulating T cell responses (Chiurchiù et al., 2016; Hong et al., 2003). Several chemically distinct molecules (RvD1‐RvD6) are biosynthesized from DHA via enzymatic action of 15‐lipoxygenase and 5‐lipoxygenase (Norling & Perretti, 2013). Resolvin D1 (RvD1) has been studied for its potential benefits in inflammatory diseases such as periodontitis and rheumatoid arthritis (Benabdoun et al., 2019; Mustafa et al., 2013; Norling et al., 2016). RvD1 played a vital role in wound healing due to the increased proliferation of periodontal ligament fibroblasts in vitro (Mustafa et al., 2013). In addition, the bone protective action of RvD1 was investigated through in vitro and in vivo experiments. RvD1 prevented bone resorption and reduced proinflammatory mediators in mice with inflammatory arthritis (Benabdoun et al., 2019). Vasconcelos et al. (2018) investigated the healing of rat femoral defects filled with porous 3D chitosan scaffolds embedded with RvD1. The results showed a more favorable bone healing response after RvD1 treatment compared to the control defects. However, little is known of the impact of RvD1 on stimulating vascular regeneration. A recent study has demonstrated that RvD1 can inhibit neointimal hyperplasia in a rabbit vein graft model (Wu et al., 2018), suggesting that RvD1 can potentially be used to prevent restenosis and bypass graft failure.

Although the application of RvD1 could inhibit bone resorption and promote bone preservation, there is a major obstacle due to its instability and short half‐life. Therefore, it is necessary to develop a suitable delivery system for local delivery of RvD1. Biodegradable biomaterials have been widely used as scaffolds in the field of BTE due to their biocompatibility, osteoinductivity, and possibilities of adjusting the mechanical strength for individual load requirements (Salgado et al., 2004). Biodegradable biomaterials include natural polymers such as chitosan, collagen, elastin, alginate, gelatin, and hyaluronic acid or synthetic polymers such as polycaprolactone, polylactic‐co‐glycolic acid, and polylactic acid (Bharadwaz & Jayasuriya, 2020). These scaffolds can be applied as carriers for the targeted delivery of drugs, growth factors, genes, or cells (Lutolf & Hubbell, 2005).

In this study, biocompatible scaffolds, collagen 3D Nanopore scaffold (COL) and Pluronic F127 (F127) approved by the Food and Drug Administration (FDA), were used as carriers to deliver RvD1 into calvarial defect sites. Collagen is the main extracellular matrix component in mammals and has been widely used for tissue engineering as it contains amino acid sequences for cell bio‐recognition (Ferreira et al., 2012). F127 is a widely used thermoresponsive polymer whose solution becomes hydrogel at a temperature above the lower critical gelation temperature (Gioffredi et al., 2016). It has been applied for delivery of cells and drugs in previous studies (Brunet‐Maheu et al., 2008; Guzmán et al., 1992).

Several tissue‐engineering approaches to the reconstruction of bony defects have been studied, but the effects of RvD1 on promoting bone and vascular regeneration have not been extensively evaluated. Therefore, the purpose of this study was to investigate if a local delivery of RvD1 can stimulate angiogenesis and osteogenesis in a rat calvarial defect model.

## 2 MATERIALS AND METHODS

### 2.1 Cell culture and cell viability

The human osteoblastic osteosarcoma cell line MG63 cells (CL‐0157, Procell, Wuhan, China) were cultured in MEM medium (Procell) supplemented with 10% fetal bovine serum (FBS; Abwbio) at 37°C in a high humidity environment containing 5% CO_2_. The medium was replaced every 2 days. MG63 cells with 80% confluence were trypsinized from culture flasks.

Cell viability was measured with the Cell Counting Kit‐8 (CCK‐8) assay (AbMole). Briefly, MG63 cells were seeded at a density of 1 × 10^3^ cells/well in a 96‐well plate and cultured in 100 μL of MEM medium containing FBS for 24 h. To examine the effect of RvD1 (Cayman Chemical) on cell viability, MG63 cells were treated with RvD1 (10 ng/ml, 100 ng/ml) for 24 and 48 h. Untreated cells served as control. After treatment, 10 μL CCK‐8 was added to each well, and cells were incubated for another 2 h with 5% CO_2_ at 37°C. Then, absorbance was read at 450 nm using an infinite 200Pro microplate reader (Tecan). The experiment was performed in sextuplicate. The control group was defined as 100% cell viability, and other groups were normalized to this value.

### 2.2 Scaffold preparation

COL with a pore size of 150 μm was purchased from VivaCell Bioscience, and F127 powder was obtained from Solarbio. Round COL, 5 mm in diameter, was prepared by a sterile dermal punch. 30% (w/v) F127 solution was made by dissolving F127 powder in phosphate‐buffered saline (PBS) and then put in a constant temperature shaker at 4°C overnight to ensure the complete dissolution of F127 powder. The prepared solution was stored at 4°C in the refrigerator. Before implantation, RvD1 was added to the F127 solution with a 5 ng/μL final concentration. Then 20 μL F127 solution with or without RvD1 was placed on the surface of COL, and the constructs were allowed to set at 37°C before surgery until the F127 solution became hydrogel.

### 2.3 Surgery and transplantation procedure

All animal experiments were approved by the Ethical Committee of the School of Stomatology, Lanzhou University (approval number LZUKQ‐2020‐022) and performed in strict accordance with the guidelines of Laboratory Animal Care of Lanzhou University. A total of 26 female Sprague‐Dawley (SD) rats (180–200 g), purchased from the Experimental Animal Center of Lanzhou University, were used as recipients to establish calvarial defects. All rats were kept in an appropriate environment for 1 week before surgery and freely obtained standard chow and water. 26 rats were divided into three different groups: symmetrical defects of 6 rats were not treated (Empty group); symmetrical defects of 10 rats were filled with COL and F127 incorporated with RvD1 (RvD1‐COL‐F127 group), and symmetrical defects of 10 rats were filled with COL and F127 (COL‐F127 group).

The surgical procedure is described elsewhere (Xing et al., 2011). Briefly, the rats were anesthetized and the site was shaved and disinfected, then a midline incision was made through the skin and periosteum to expose the calvaria. Symmetrical bone defects, 5 mm in diameter, were created using a trephine bur with constant physiologic saline irrigation. During the procedure, special care was taken to avoid damage to the dura mater. Absorbable sutures were applied for wound closure. After implantation, RvD1 (100 ng in 20 μL PBS) was administrated subcutaneously every 7 days for 4 weeks in the RvD1‐COL‐F127 group. The other groups were treated with the same volume of PBS. Half of the rats were sacrificed at weeks 1 and 8 post‐implantation, and the calvarias were retrieved for evaluation. Pictures were captured using a digital camera (Nikon).

### 2.4 Radiographic analysis

Before decalcification, radiographs of the samples harvested at week 8 were taken using a dental X‐ray machine (Dentsply Sirona). Exposure conditions were 7 mA, 0.03 s, and 70 kV, and the X‐ray beam was perpendicular to the bone defect areas that were parallel to the ground when the radiographic images were taken. To quantitatively determine the whole area of the newly formed osteoid, a common threshold was applied to include all high‐density areas. The osteoid area of each defect and the bone defect areas (*n* = 10) were determined, and the area fraction of the newly formed osteoid was calculated by the Image J software (National Institutes of Health). After radiological analysis, each sample was separated in half for histological analysis and gene analysis, respectively.

### 2.5 Histological analysis

Tissue samples at weeks 1 and 8 intended for histological evaluation were fixed with 4% paraformaldehyde for 24 h, decalcified in 10% EDTA solution for 4 weeks at room temperature, and dehydrated in a graded alcohol series. The samples were embedded in paraffin and sliced into 4‐μm‐thick sections using a pathological slicer (Leica RM2016, Wetzlar, Germany). Sections were stained with hematoxylin and eosin (HE) to quantify the number of inflammatory cells and the area of new bone formation using Image J. At week 1, average numbers of inflammatory cells were calculated from the counts of three random images at 40× magnification in each sample (*n* = 5) in the RvD1‐COL‐F127 and COL‐F127 groups. At week 8, the area of the newly formed osteoid and the bone defect areas were determined (*n* = 6). Osteoid area fraction was defined as the ratio of the area of the newly formed osteoid to the bone defect areas.

### 2.6 Tartrate‐resistant acid phosphatase (TRAP) staining

Osteoclasts were stained using a TRAP‐hematoxylin counterstaining protocol. Briefly, sections were deparaffinized, rehydrated with graded alcohol, and subjected to TRAP staining. TRAP incubation solution (G1050, Servicebio) was prepared according to the manufacturer's instructions. Sections were incubated in distilled water at 37°C for 2 h; TRAP incubation solution was dropped on each section and incubated at 37°C for 20 min. Then, sections were counterstained with hematoxylin and mounted with neutral gum. The images were obtained by a light microscope (BX53, Olympus). Average TRAP‐positive cells were calculated from the counts of five random images at 40× magnification in each sample (*n* = 6) in the RvD1‐COL‐F127 and COL‐F127 groups.

### 2.7 Immunohistochemistry

CD31 staining was used to visualize and quantify newly formed blood vessels. Sections were deparaffinized, rehydrated, and incubated in antigen retrieval buffer. 3% hydrogen peroxide was used to block endogenous peroxidase activity. After washing three times with PBS, sections were incubated with 3% BSA for 30 min at room temperature to block non‐specific binding. Sections were incubated with the primary antibody against CD31 (GB11063‐2, Servicebio) diluted at 1:1000 overnight at 4°C, followed by HRP‐conjugated goat anti‐rabbit IgG (G1215, Servicebio) diluted 1:200 for 50 min at room temperature. After washing three times with PBS, a diaminobenzidine solution (G1211, Servicebio) was used to visualize the antibody complex. Finally, counterstaining was performed in hematoxylin staining solution for 3 min, followed by mounting with neutral gum. Blood vessels were identified by their luminal structure and the presence of red blood cells. The number of newly formed vessels was quantified from seven sections per group in the RvD1‐COL‐F127 and COL‐F127 groups.

### 2.8 RNA isolation and real‐time reverse transcription‐polymerase chain reaction (RT‐PCR) in vivo

Tissue samples at week 1 intended for RT‐PCR were kept in RNA Later (Invitrogen) overnight. Total RNA was extracted using TRIzol Reagent (Ambion) according to the manufacturer's instructions. RNA purity and quantification were measured by UV5Nano (Mettler Toledo GmbH). The reverse transcription reaction was carried out using EasyQuick RT MasterMix from Cwbiotech on a LifeECO PCR machine (Bioer). The primers of rat genes, including tumor necrosis factor‐α (TNF‐α), interleukin 4 (IL‐4), interleukin 1β (IL‐1β), vascular endothelial growth factor (VEGF), alkaline phosphatase (ALP), receptor activator of nuclear factor‐κB ligand (RANKL) and glyceraldehyde‐3‐phosphate dehydrogenase (GAPDH) were obtained from Invitrogen to detect mRNA levels. Primer sequences were listed in Table 1. RT‐PCR reaction was performed using UltraSYBR Mixture (Cwbiotech), and mixtures were prepared for each target cDNA according to the manufacturer's instructions. Amplification was conducted in a Rotor‐Gene Q PCR machine (Qiagen, Hilden, Germany). The data were analyzed using a 2^−ΔΔCt^ method, and GAPDH was used as an endogenous control.

**TABLE 1:** Primer sequences used for real‐time reverse transcriptase‐polymerase chain reaction

Gene	Primer sequence (5′–3′)
TNF‐α	F: AGAACTCAGCGAGGACACCAAG
	R: TGTATGAGAGGGACGGAACCT
IL‐1β	F: AGGCTGACAGACCCCAAAAG
	R: GGTCGTCATCATCCCACGAG
IL‐4	F: CGTGATGTACCTCCGTGCTT
	R: ATTCACGGTGCAGCTTCTCA
VEGF	F: ACCATGCCAAGTGGTGAAGT
	R: GCTGGCTTTGGTGAGGTTTG
ALP	F: TGCAGGATCGGAACGTCAATTA
	R: AAATGAGTTGGTAAGGCAGGGT
RANKL	F: AGATGCGACGTACTTTGGGG
	R: CGAGAGAGGACCGTGAGTTT
GAPDH	F: ACTCCCATTCTTCCACCTTTG
	R: CCCTGTTGCTGTAGCCATATT

Abbreviations: F, Forward; R, Reverse.

### 2.9 Statistical analysis

GraphPad Prism was used for statistical processing and analysis. All quantitative results were presented as mean ± standard deviation (SD). A *t*‐test was performed to compare data from the RvD1‐COL‐F127 and COL‐F127 groups. A value of *p* < 0.05 was considered statistically significant.

## 3 RESULTS

### 3.1 The effect of RvD1 on cell viability

**FIGURE 1:**
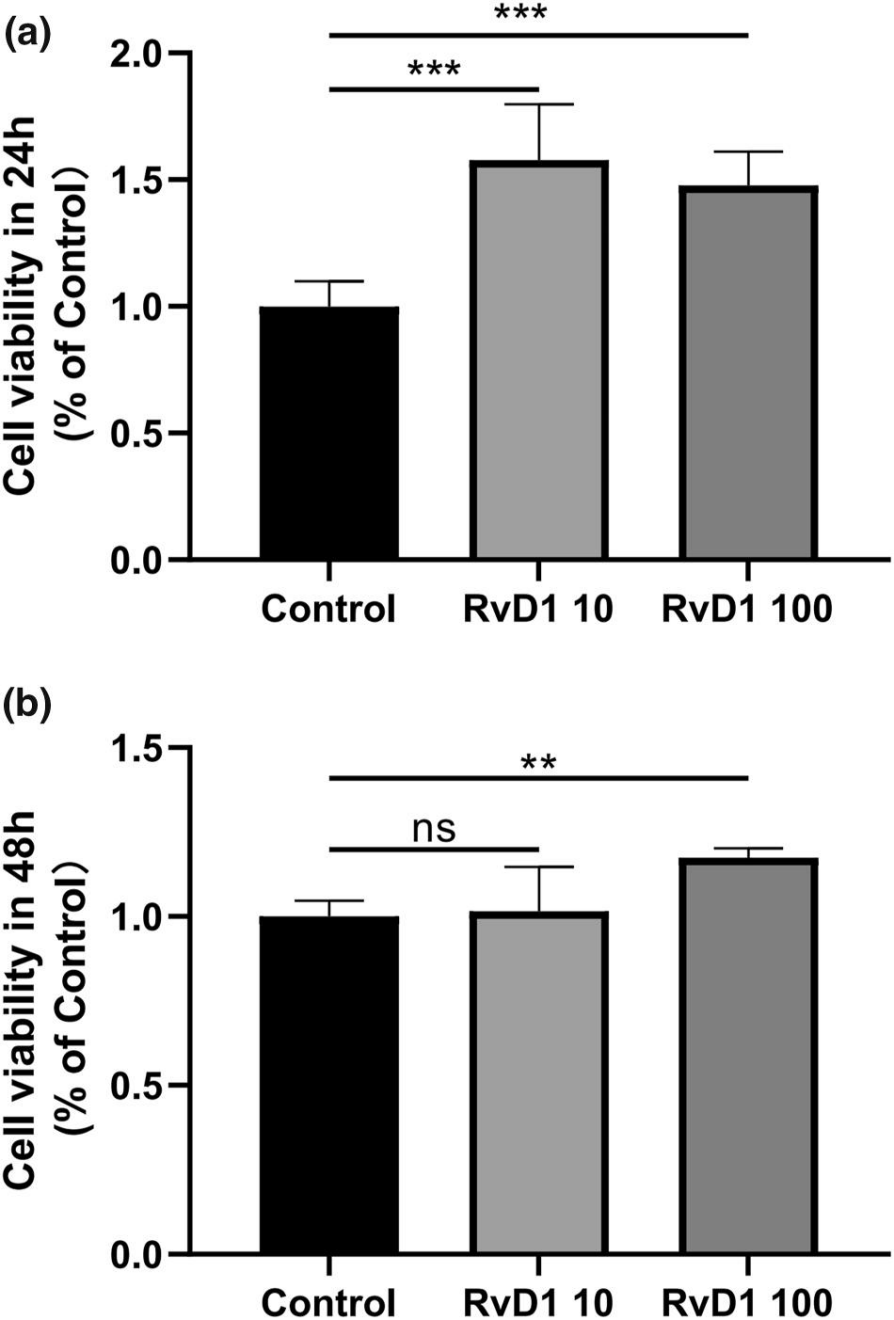
RvD1 did not impair MG63 cells viability. MG63 cells were incubated with RvD1 (10 ng/ml, 100 ng/ml) for 24 h (a) and 48 h (b). The effect of RvD1 on cell viability was evaluated by the Cell Counting Kit‐8 assay. Control, cells without treatment. **p < 0.01, ***p < 0.001. Resolvin D1, RvD1

As shown in Figure 1, the in vitro data revealed that RvD1 neither alter the cell viability nor induce cytotoxicity in MG63 cells compared with the control group. On the contrary, RvD1 significantly increased cell viability after 24 h for both doses and after 48 h for the higher dose used.

### 3.2 Radiographic analysis

**FIGURE 2:**
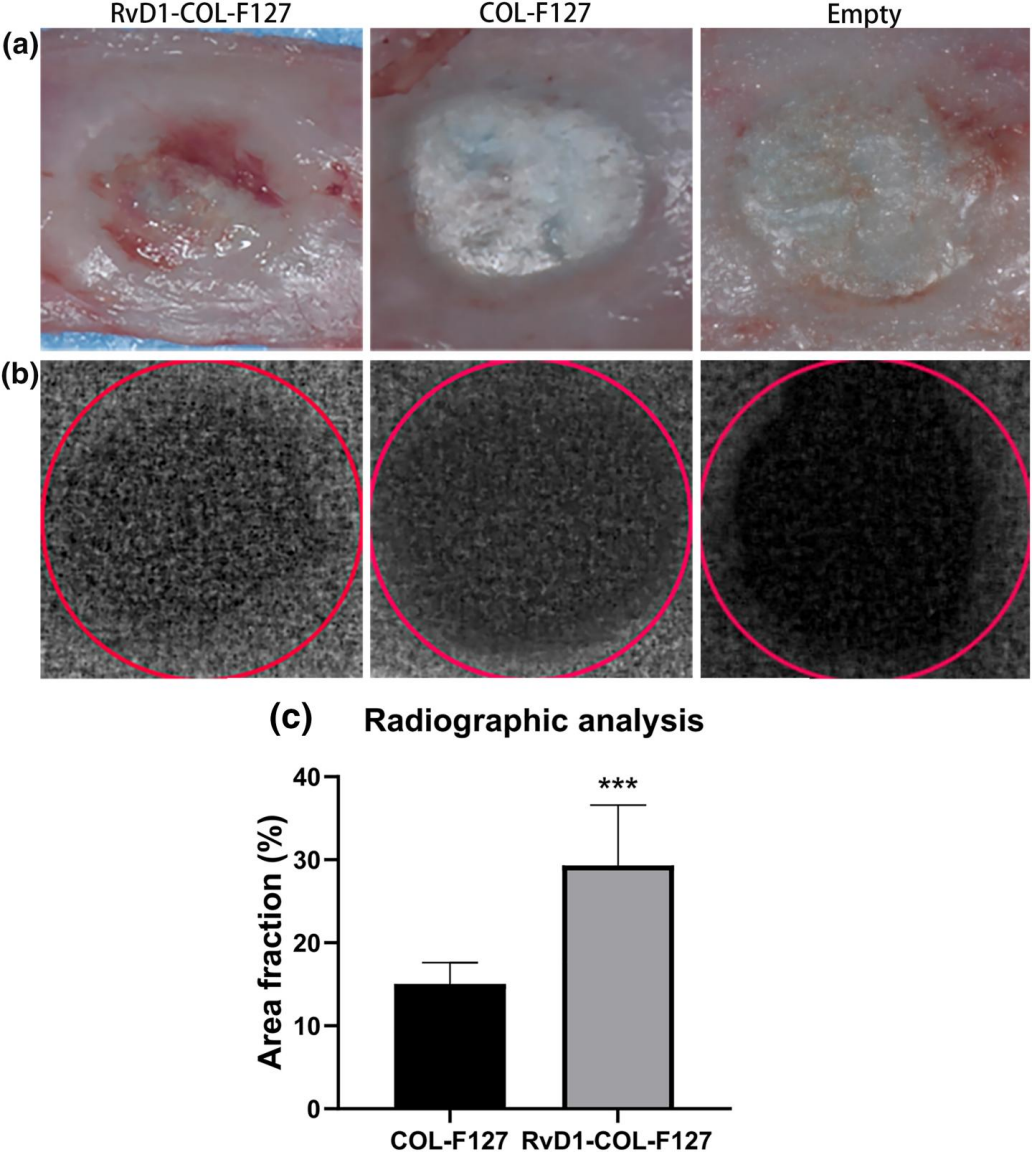
Macroscopic view (a) and radiographic pictures (b) were obtained from rat calvarias at week 8 after implantation. Scale bar (a): 5 mm. The red circle in figure B represented the edge of the 5‐mm calvarial defects. (c) The radiographic results showed that the area fraction of osteoid was higher in the RvD1‐COL‐F127 group than that in the COL‐F127 group. ***p < 0.001

Macroscopic views (Figure 2a) of rat calvarias were obtained at week 8 after implantation from different groups. Small areas with high density could be observed in the COL‐F127 and Empty groups. However, most samples from the RvD1‐COL‐F127 group showed larger areas with high density than the other groups (Figure 2b). The radiographic results showed that osteoid area fraction (mean ± SD; *n* = 10) was (29.3 ± 7.3)% in the RvD1‐COL‐F127 group compared to (14.0 ± 2.4)% for the COL‐F127 group, therefore, the percentage of newly formed osteoid was higher in the RvD1‐COL‐F127 group than that in the COL‐F127 group (Figure 2c).

### 3.3 Histological analysis

**FIGURE 3:**
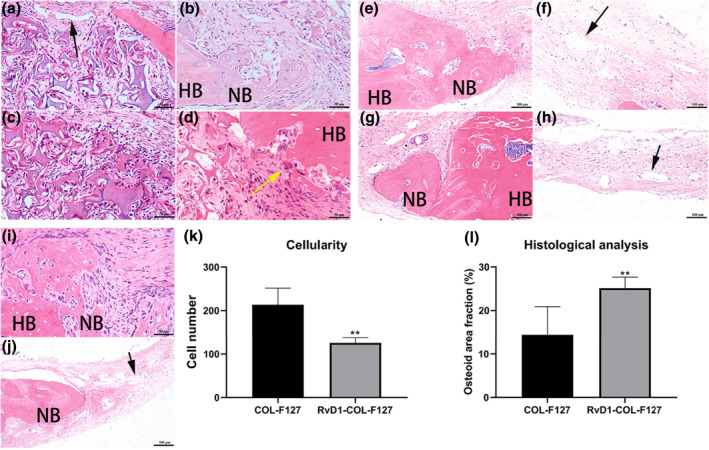
Hematoxylin and eosin (HE) staining results. At week 1, soft tissue grew into bone defect areas in the RvD1‐COL‐F127 (a, b), COL‐F127 (c, d) and Empty groups (i), a variety of cells such as inflammatory cells were observed around degraded collagen materials. Yellow arrows indicated osteoclasts. Scale bar (a, b, c, d, i) 50 μm. At week 8, osteoid was formed around the host bone in the RvD1‐COL‐F127 (e), COL‐F127 (g), and Empty groups (j). Host bone, HB; new bone, NB. Scale bar (e, g, j) 100 μm. Newly formed blood vessels appeared in the internal part of the defect areas in the RvD1‐COL‐F127 (f), and COL‐F127 groups (h). Black arrows indicated blood vessels. Scale bar (f, h): 100 μm. (k) Quantification of infiltrating inflammatory cells. Treatment with resolvin D1 alleviated inflammatory cell infiltration surrounding degraded collagen scaffolds at week 1. **p < 0.01. (l) Quantitative analysis of newly formed osteoid. The results showed enhanced osteoid formation in the RvD1‐COL‐F127 group compared with the COL‐F127 group. The osteoid area fraction represented the ratio of the total area of osteoid formation to the total area of calvarial defects. **p < 0.01

Soft tissue grew into calvarial defect areas at week 1 after implantation in all groups. Various cell populations such as fibroblastic cells and inflammatory cells were observed within the defects (Figure 3a–d,i). The number of inflammatory cells in the COL‐F127 group was higher than that in the RvD1‐COL‐F127 group (Figure 3k). Degradation of grafting materials was observed in both the RvD1‐COL‐F127 and COL‐F127 groups.

The sections were stained with HE to evaluate the osteoid formation within the defect sites after 8 weeks of implantation. The disappearance of grafting materials was accompanied by the formation of fibrous tissue. In all groups, osteoid‐like tissue was formed from the periphery of the host bone, and the defect areas were mostly filled with fibrous tissue (Figure 3e,g,j). In addition, newly formed blood vessels were also observed in the defect sites (Figure 3f,h). The quantitative results showed that an increase in osteoid‐like tissue was observed in the RvD1‐COL‐F127 group, while only a small quantity of osteoid‐like tissue was visible in the COL‐F127 group (Figure 3l). Histological results were in line with the radiographic analysis showing extensive osteoid‐like tissue formed in the RvD1‐COL‐F127 group.

### 3.4 Osteoclast activity

**FIGURE 4:**
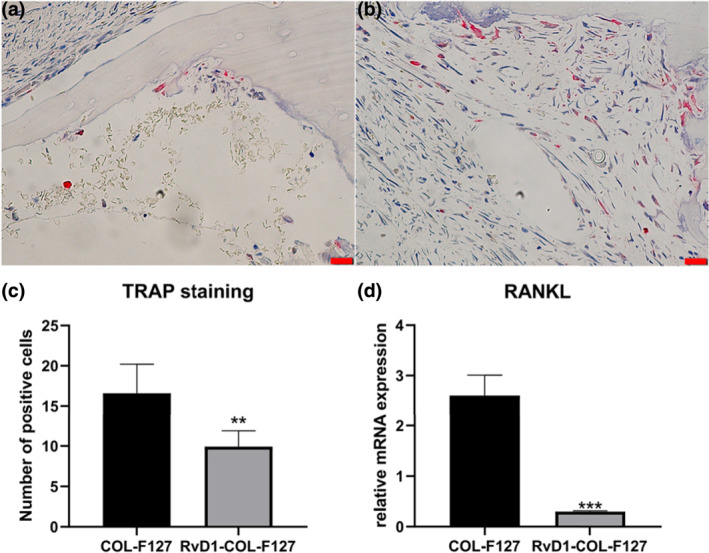
Representative images of TRAP staining at the defect sites at week 1 after implantation. TRAP‐positive osteoclasts were observed in the RvD1‐COL‐F127 (a) and COL‐F127 (b) groups, which were located at the surface of the host bone. The red color is indicative of TRAP activity. Scale bar: 50 μm. (c) More TRAP‐positive cells were observed in the COL‐F127 group compared with the RvD1‐COL‐F127 group. **p < 0.01. (d) The results from RT‐PCR showed that RANKL expression was higher in the COL‐F127 group than that in the RvD1‐COL‐F127 group at week 1. ***p < 0.001. Tartrate‐resistant acid phosphatase, TRAP; real‐time reverse transcription‐polymerase chain reaction, RT‐PCR; receptor activator of nuclear factor‐κB ligand, RANKL

TRAP staining revealed that osteoclasts were present at the surface of the host bone at week 1 after implantation (Figure 4a,b). TRAP‐positive cells were decreased in the RvD1‐COL‐F127 group treated with RvD1 after 1 week compared with the COL‐F127 group (Figure 4c). The gene expression of RANKL in the COL‐F127 group was higher than that in the RvD1‐COL‐F127 group at week 1 (Figure 4d).

### 3.5 Quantification of newly formed blood vessels

**FIGURE 5:**
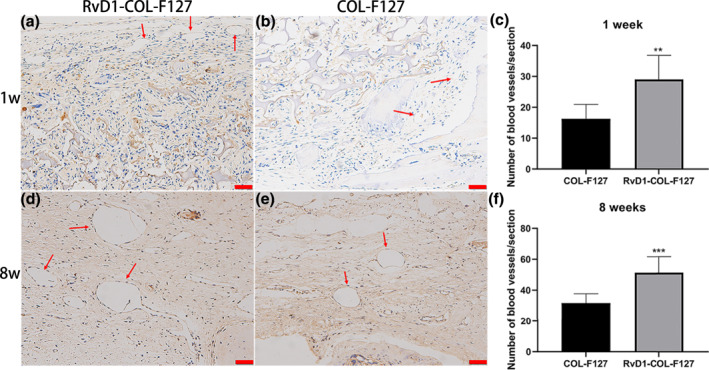
CD31 staining of sections at weeks 1 and 8 after implantation. Newly formed blood vessels indicated by red arrows were observed from the RvD1‐COL‐F127 (a, d) and COL‐F127 groups (b, e). Scale bar: 100 μm. (c, f) The total number of newly formed blood vessels at weeks 1 and 8 after implantation was recorded in the RvD1‐COL‐F127 and COL‐F127 groups. *p < 0.05

CD31 staining was performed to characterize the newly formed blood vessels in the defect sites after post‐operatively 1 week and 8 weeks. At week 1, the blood vessels were distributed around the defect sites (Figure 5a,b). After 8 weeks of implantation, blood vessel structures appeared in the internal part of the defect sites (Figure 5d,e). The number of blood vessels in the RvD1‐COL‐F127 group was higher than that in the COL‐F127 group at each time point, and the number of blood vessels in both groups at week 8 increased compared with week 1 (Figure 5c,f).

### 3.6 Gene expressions from in vivo experiments

**FIGURE 6:**
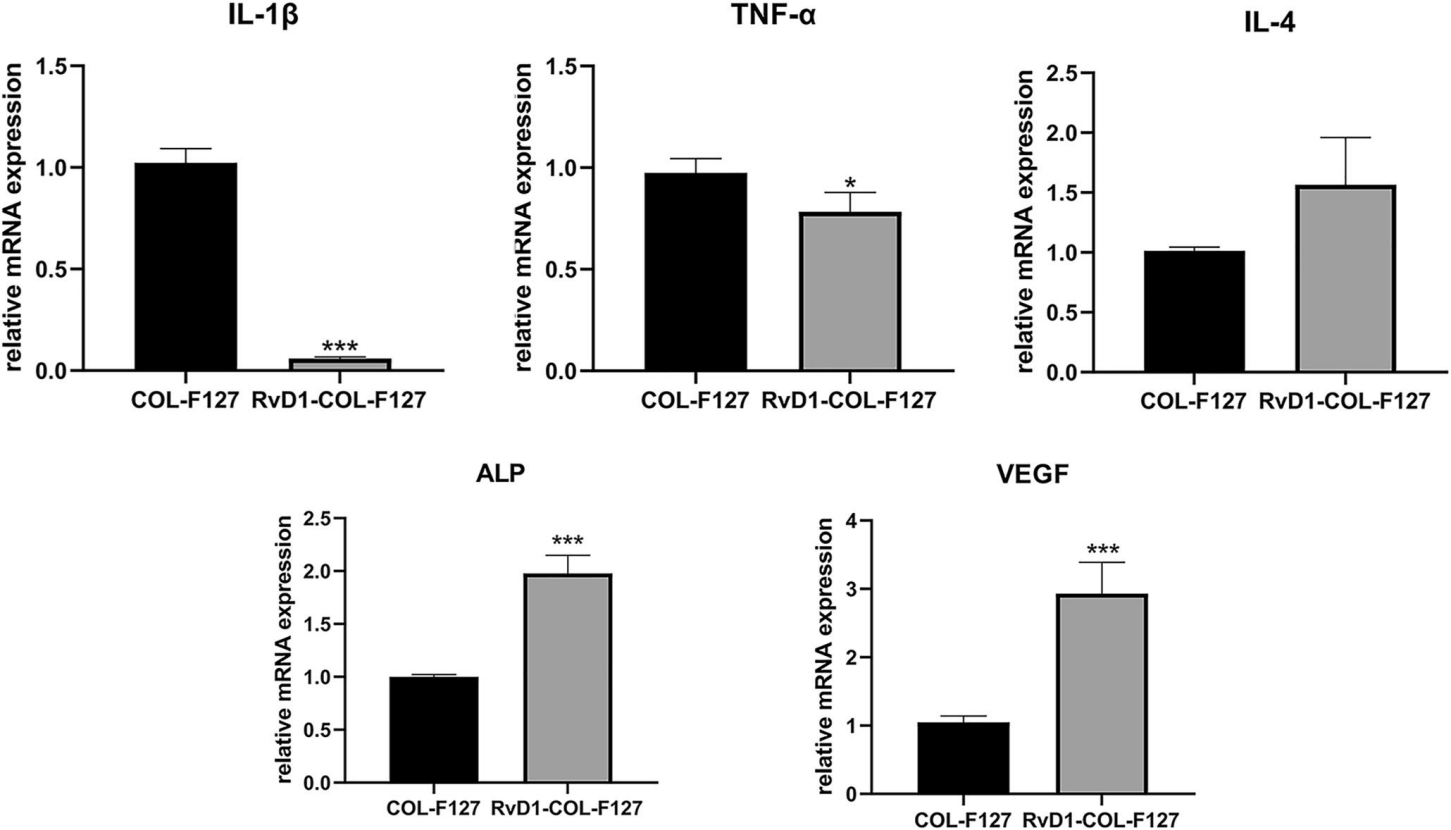
Real‐time reverse transcription‐polymerase chain reaction results from the RvD1‐COL‐F127 and COL‐F127 groups after 1 week. Significant higher expression of alkaline phosphatase (ALP) and vascular endothelial growth factor (VEGF) could be detected from the RvD1‐COL‐F127 group. However, the COL‐F127 group expressed significantly higher mRNA levels of proinflammatory markers compared to the RvD1‐COL‐F127 group. Interleukin 1β (IL‐1β), tumor necrosis factor‐α (TNF‐α), interleukin 4 (IL‐4). Data are expressed as the mean ± SD. *p < 0.05; ***p < 0.001

At week 1, expression levels of IL‐1β and TNF‐α were downregulated in the RvD1‐COL‐F127 group, which suggested decreased inflammatory responses in calvarial defect areas with RvD1 treatment at week 1. There was a higher mRNA expression level of IL‐4 in the RvD1‐COL‐F127 group compared with the COL‐F127 group, although the difference was not statistically significant. Expression levels of ALP and VEGF increased in the RvD1‐COL‐F127 group compared with the COL‐F127 group (Figure 6).

## 4 DISCUSSION

This study was designed to identify the impact of RvD1 on bone regeneration using a calvarial defect model. RvD1 could attenuate inflammatory responses at week 1 and improve the microenvironment for bone and blood vessel formation. The results demonstrated that new bone formation in calvarial defect sites could be accelerated by delivering RvD1 from the first postoperative day and one time weekly for a total dose of 500 ng. At weeks 1 and 8 after implantation, tissue samples with RvD1 treatment disclosed more blood vessel formation.

Implantation of biomaterials into the body is followed by an inflammatory response, which may affect the integration of the graft and tissue regeneration (Gower et al., 2014). Inflammatory cytokines are involved in the regulation of osteoclastogenesis and therefore directly affect bone remodeling (Ai‐Aql et al., 2008). Hence, an enhanced understanding of the inflammatory response to implanted biomaterials may help understand how adequate bone regeneration can be achieved. RvD1 can promote the resolution of inflammation with well‐known pro‐resolving and anti‐inflammatory properties. For example, it was reported that RvD1 was able to reduce lung inflammation in mice exposed to cigarette smoke (Hsiao et al., 2013). In the current study, intermittent RvD1 administration was applied to recover bone defects. At week 1 after implantation, a decreased number of inflammatory cells was observed, and proinflammatory cytokines IL‐1 and TNF‐α significantly decreased in the RvD1‐COL‐F127 group compared to the COL‐F127 group, indicating early cessation of the inflammatory responses. These findings suggested that rapid resolution of the inflammatory responses might be beneficial for bone healing.

In the present study, the results of CD31 staining showed greater numbers of newly formed blood vessels in the RvD1‐COL‐F127 group, and the expression of VEGF increased after RvD1 treatment, demonstrated by RT‐PCR. Increased levels of VEGF could be a contributing factor to the increased blood vessel formation observed after RvD1 treatment. Previous studies showed that RvD1 inhibited activation, proliferation, and migration of vascular smooth muscle cells in vitro and attenuated neointimal hyperplasia in vascular injury (Miyahara et al., 2013; Wu et al., 2018). It has also been reported that aspirin‐triggered resolvin D1 (AT‐RvD1), which possess the same anti‐inflammatory effect as RvD1 (Sun et al., 2007), enhanced endothelialization, promoted vascularization and improved smooth muscle regeneration in rat abdominal aortas (Shi et al., 2019). One of the possible mechanisms of RvD1 promoting vascularization could be the role of RvD1 on macrophage polarization. Two macrophage subtypes with different functions, including the pro‐inflammatory "M1" phenotype (classically activated) and the anti‐inflammatory "M2" phenotype (alternatively activated), have been recognized (Mantovani et al., 2013). RvD1 can switch macrophage polarization toward an M2 phenotype in adipose tissue of obese mice (Titos et al., 2011). Vasconcelos et al. (2015) demonstrated that porous 3D chitosan scaffolds incorporated with RvD1 caused a decrease in inflammatory cells and triggered a shift in the macrophage phenotype toward an M2 phenotype in vivo using a mouse air‐pouch model of inflammation. Several growth factors, including bone morphogenetic protein‐2 (BMP‐2), osteopontin, and VEGF produced by M2 macrophages, are inducers of new bone formation and blood vessels (Champagne et al., 2002; Ferrante et al., 2013; Takahashi et al., 2004). These results suggested that RvD1 could promote anti‐inflammatory M2 macrophages to produce more growth factors such as BMPs and VEGF for osteogenesis and angiogenesis.

The results from this study showed that enhanced bone formation was observed radiographically and histologically. The expression of RANKL was increased, and the mRNA level of ALP was decreased in the COL‐F127 group compared with the RvD1‐COL‐F127 group. The number of TRAP‐positive osteoclasts was also reduced in the RvD1‐COL‐F127 group. The bone protective effect of RvD1 described by our findings is consistent with previous reports. It has been demonstrated that the level of RvD1 in the synovial fluid of osteoarthritis joints was high, and RvD1 could inhibit cartilage degradation induced by osteoarthritis (Benabdoune et al., 2016). RvD1 played a key role in preventing bone resorption, suggesting that RvD1 may represent a potential therapeutic option for inflammatory arthritis (Benabdoun et al., 2019). In tissue engineering, porous 3D chitosan scaffolds embedded with RvD1 were beneficial for bone repair in rat femoral defects, with a role in switching macrophage polarization (Vasconcelos et al., 2018). In the context of experimental periodontitis, resolvin D2 treatment inhibited bone loss by decreasing RANKL expression and increasing osteoprotegerin expression (Mizraji et al., 2018). Resolvin E1 also prevented alveolar bone loss and inhibited osteoclastogenesis (Hasturk et al., 2006, 2007; Lee et al., 2016). Therefore, these results suggested that RvD1 may prevent bone resorption via inhibiting osteoclast differentiation and activation. Further in vitro investigation is warranted into the molecular mechanisms underlying the action of RvD1 on osteoblasts and osteoclasts.

## 5 CONCLUSIONS

In summary, our results demonstrated a role for RvD1 in controlling the inflammatory microenvironment and promoting bone healing and angiogenesis in a rat calvarial defect model.

## AUTHOR CONTRIBUTIONS

Xiaofeng Jiang: design of the experiments, performing the experiments, data analysis, writing the manuscript. Jing Liu: performing in vivo experiments, writing the manuscript, literature review. Si Li: performing in vivo experiments, writing the manuscript. Yingfei Qiu: performing in vitro experiments, literature review. Xiaoli Wang: data analysis, editing the manuscript. Xiaoli He: data analysis, editing the manuscript. Torbjørn Ø. Pedersen: revision of the manuscript, editing the manuscript. Kamal Mustafa: optimal design of the experiments, editing the manuscript. Ying Xue: correspondence, optimal design of the experiments, supervision of the experiments. Manal Mustafa: data analysis, revision of the manuscript. Alpdogan Kantarci: revision of the manuscript. Zhe Xing: correspondence, conceptualization, supervision of the experiments, financial support. All authors read and approved the final manuscript.

## CONFLICT OF INTEREST

The authors declare that they have no competing interests.

## Data Availability

The data that support the findings of this study are available from the corresponding author upon reasonable request.
